# Inducing T cell dysfunction by chronic stimulation of CAR-engineered T cells targeting cancer cells in suspension cultures

**DOI:** 10.1016/j.xpro.2022.101954

**Published:** 2023-01-04

**Authors:** Mehmet Emrah Selli, Jack H. Landmann, Corvin Arveseth, Nathan Singh

**Affiliations:** 1 Washington University School of Medicine, Division of Oncology, St. Louis, MO 63105, USA

**Keywords:** Cancer, Immunology, Biotechnology and bioengineering

## Abstract

Several pre-clinical models reveal that chronic chimeric antigen receptor (CAR) stimulation drives a dysfunctional state that mimics *in vivo* failure. In this protocol, we describe steps to induce T cell dysfunction by persistent and long-term stimulation of CAR-engineered T cells using antigen-expressing cancer cells in suspension cultures. We first described a validated method for manufacturing of CAR T cells, followed by a detailed method for chronic stimulation of CAR T cells and a strategy to evaluate these cells during the process of chronic stimulation.

For complete details on the use and execution of this protocol, please refer to Singh et al. (2020).^1^

## Highlights

• Production of chimeric-antigen-receptor (CAR)-expressing human T cells
• Directing CAR T cells to become dysfunctional through chronic stimulation
• Downstream analysis of CAR T cells during various stages of chronic stimulation

## Abstract (editor highlights)

Publisher’s note: Undertaking any experimental protocol requires adherence to local institutional guidelines for laboratory safety and ethics.

## Graphical abstract

**Figure:**
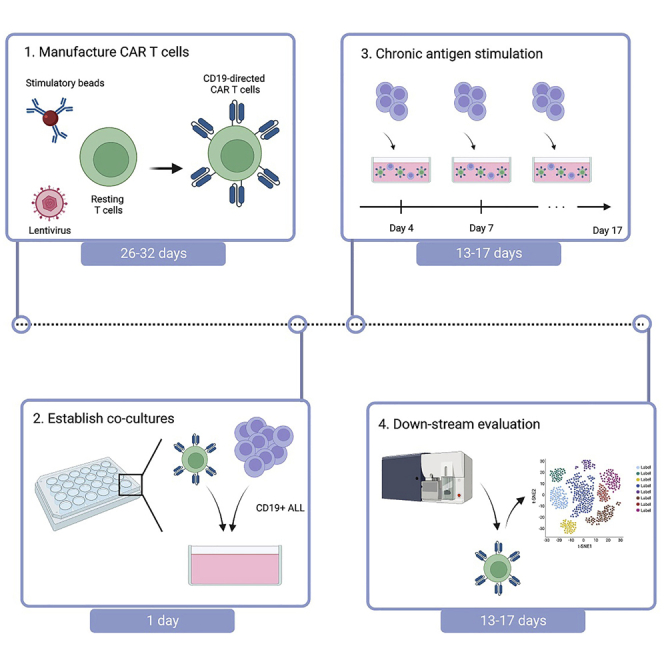

## Before you begin

Chronic T cell receptor stimulation is the mechanism that drives T cell exhaustion. Based on clinical observations that seemingly “fit” CAR-engineered T cell products can fail to control hematologic cancers in patients^2^^,^^3^ and that higher disease burden increases the likelihood of therapeutic failure,^4^ several groups have investigated the impact of persistent CAR stimulation. These studies have consistently demonstrated that chronic CAR activation, either through tonic signaling^5^^,^^6^ or via engagement with target antigen^1^^,^^7^ drives the onset of T cell dysfunction that mimics clinical CAR T cell failure. As such, these models can serve as the basis for mechanistic studies aimed at identifying the molecular drivers of CAR-driven dysfunction and correlating biology. The protocol below articulates how to establish, maintain and analyze chronic stimulatory cultures of primary human T cells that target malignant hematologic cancers. Specifically, we describe interrogation of T cells engineered to express CD19-targeted chimeric antigen receptors (CARs) that are chronically stimulated by the CD19+ acute lymphoblastic leukemia (ALL) cell line Nalm6. We have performed similar chronic stimulation co-cultures using CAR T cells targeted to other blood cell antigens (i.e., CD22, CD33) and T cells engineered to express transgenic T cell receptors (TCRs) targeted to tumor-associated peptide-MHC antigens (i.e., anti-NYESO1_157-165_ TCR combined with Nalm6 engineered to express HLA-A2∗01, NYESO1 and costimulatory ligands). Each receptor:antigen pair will have unique kinetics to maintain persistent stimulation, but similar priniciples and experimental techniques apply to all.

Before you begin:

1. Thaw and expand Nalm6.
  a. Depending on the number of conditions being evaluated, ensure sufficient Nalm6 cells for the duration of the experiment.

***Note:*** In this protocol we will add 8× as many Nalm6 cells as we have T cells (effector:target ratio of 1:8), every other day. Plan ahead and have plenty of Nalm6 cells in reserve.

***Note:*** Target cell lines are maintained at optimal concentrations to ensure robust cell growth (i.e., 1 × 10^6^ cells/mL for Nalm6).

2. Prepare culture media.
  a. Our standard media for primary human T cell studies is RPMI 1640 + 10% fetal bovine serum + 1% penicillin/streptomycin + 1% HEPES (R10), see materials and equipment.

***Note:*** All incubations are performed at 37°C in 5% ambient CO_2_.

## Key resources table

**Table:** 

REAGENT or RESOURCE	SOURCE	IDENTIFIER
**Antibodies**		

PE-labeled Monoclonal Anti-FMC63 scFv Antibody, Mouse IgG1 (Y45)	Acro Biosystems	Cat# FM3-HPY53; RRID: AB_2921284

**Experimental models: Cell lines**		

Human: Nalm6, clone G5	ATCC	Cat# CRL-3273
Human: HEK-293T cells	ATCC	Cat# CRL-1573

**Recombinant DNA**		

Lentiviral packaging plasmids (encoding genes for reverse transcriptase, Gag, Rev and envelope proteins). Ours are purchased from GenScript Biotech, but any version of these third-generation plasmids will do.		

**Software and algorithms**		

FlowJo v10	BD Biosciences	https://www.flowjo.com/solutions/flowjo

**Other**		

150 mL bottle top vacuum filters, 45 μm pore size	Corning	Cat# 430627
Cell counter		
Tabletop centrifuge for 50 mL tubes		
Cell incubator (set to 37°C, 5% CO_2_)		
DynaMag-2 magnetic stand	Thermo Fisher Scientific	Cat# 12321D
Flow cytometer		
Dead Cell Removal Kit	Miltenyi Biotec	Cat# 130-090-101
CD10 MicroBead Kit, human	Miltenyi Biotec	Cat# 130-093-452
MACS cell separator	Miltenyi Biotec	Variety of options
RPMI 1640 Medium	Fisher Scientific	Cat# 11875085
HEPES (1 M)	Thermo Fisher Scientific	Cat# 15630080
L-Glutamine (200 mM; 100×)	Thermo Fisher Scientific	Cat# 25030081
Penicillin–streptomycin (10,000 U/mL)	Thermo Fisher Scientific	Cat# 15140122
Fetal bovine serum, US origin	Seradigm	Cat# 97068-085
DMEM medium, no glucose	Fisher Scientific	Cat# 11966025
Opti-MEM I Reduced Serum Medium, GlutaMAX Supplement	Thermo Fisher Scientific	Cat# 51985091
DPBS, no calcium, no magnesium	Thermo Fisher Scientific	Cat# 14190136
0.05% Trypsin-EDTA	Thermo Fisher Scientific	Cat# 25300054
Lipofectamine 3000 Transfection Reagent	Thermo Fisher Scientific	Cat# L3000150
CTS DynaBeads CD3/CD28	Gibco	Cat# 40203D

## Materials and equipment

**Table:** Complete RPMI (R10)

Reagent	Final concentration	Amount
RPMI 1640 Medium	N/A	870 mL
Fetal Bovine Serum	10% (v/v)	100 mL
HEPES (1 M)	10 mM	10 mL (1%)
Penicillin–Streptomycin (10,000 U/mL)	100 U/mL	10 mL (1%)
L-Glutamine (200 mM; 100×)	2 mM; 1×	10 mL (1%)
**Total**	**N/A**	**1 L**

Filter sterilize R10 through a 22 μm filter. Store at 4°C for upto 4 weeks.

**Table:** Complete DMEM (D10)

Reagent	Final concentration	Amount
DMEM Medium, no glucose	N/A	870 mL
Fetal Bovine Serum	10% (v/v)	100 mL
HEPES (1 M)	10 mM	10 mL (1%)
Penicillin–Streptomycin (10,000 U/mL)	100 U/mL	10 mL (1%)
L-Glutamine (200 mM; 100×)	2 mM; 1×	10 mL (1%)
**Total**	**N/A**	**1 L**

**Table:** Flow cytometry buffer (FACS buffer)

Reagent	Final concentration	Amount
DPBS, no calcium, no magnesium	N/A	1 L
Fetal Bovine Serum	3% (v/v)	30 mL

Store at 4°C for up to 4 weeks.

## Step-by-step method details

### Part I: Production of lentiviral vectors

**Timing: 10–14 days**

In this step, lentiviral vectors carrying the CAR transgene are prepared, concentrated and titrated to determine viral vector concentration.

***Note:*** The viral products have been made replication-defective by isolation of packaging constructs (Rev, Gag/Pol/RRE, VSV-G) onto three separate plasmids (third-generation), greatly reducing the likelihood of recombination events that may result in replication-competent virus. However, these products still have a theoretical potential to cause DNA damage and thus should be handled with caution. All materials used in the production and handling process should be disposed of appropriately under BSL-2+ conditions.

***Note:*** We have titrated the DNA:Lipofectamine ratio for our packaging and target plasmids. For other plasmids, it is possible that this ratio may not be most efficient for the production of lentiviral particles, and hence, further titration may be needed.

1. Thaw HEK-293T cells using standard methods.
2. Plate 3 × 10^6^ cells per T175 culture flask.

***Note:*** All volumes and methods are described for this culture size.

**Figure 1:**
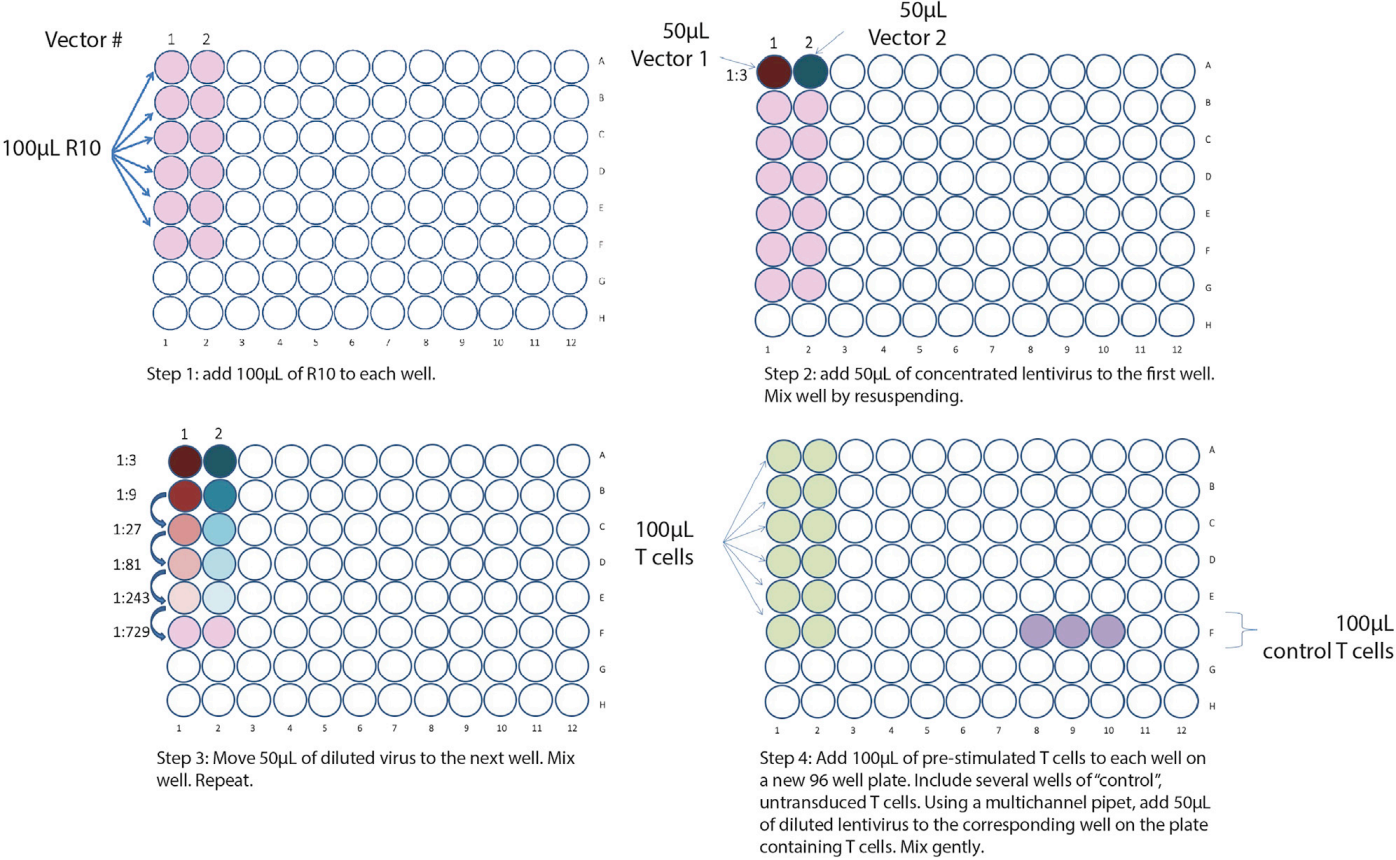
Schematic for setting up lentiviral titer using human T cells
  o. Add 50 μL of each diluted vector from the dilution plate prepared in step 15l to the corresponding wells of the titration plate containing 100 μL of T cells.
  p. Mix well by pipetting.
  q. Add 50 μL R10 to each non-transduced control well.
  r. Incubate the plate for at 37°C for 72 h.
  s. After 72 h, remove the beads:
    i. Mix each well gently and transfer the contents of each well into 1.5 mL microcentrifuge tubes.
    ii. Place the tubes onto the DynaMag-2 magnet and incubate for 1 min.
    iii. Keep the tube on the magnet and transfer the bead-free T cells to a U-bottom 96-well plate, being careful not to disrupt the beads.
  t. Add 100 μL FACS buffer into each well and centrifuge the plates at 350 × *g* for 5 min.
  u. Analyze the efficacy (%) of transduction for each titered well by flow cytometry.
    ***Note:*** Proper titration and detection of the CAR transgene requires a well-validated detection antibody. This will vary depending on the CAR. In our case, we routinely use an antibody detecting FMC63 (refer to the key resources table) to detect our FMC63-based anti-CD19 CAR.
  v. Use the following formula to calculate the amount of lentiviral particles (transducible units (TU)) per mL of concentrated virus^8^:
    i. TU/mL = (% positive cells/100) × 10^5^ × 16.6 × dilution factor.
        10^5^ is the number of T cells in each well.
        16.6 is the volume of lentivirus in each well (50 μL diluted in 100 μL in the dilution plate, then 50 μL [1/3 volume] transferred to the titer plate).
    ii. For example: if 15% of cells are positive at the 1:27 dilution, the titer would be:
      (15/100) × 10^5^ × 16.6 27 = 8.1 × 10^6^ TU/mL.
  w. Generate a graph of sample dilution (x-axis) vs. sample titer (y-axis) for each vector.
    ***Note:*** It is important to remember that this titer is an estimate of the transducible units (TU)/mL.
    ***Note:*** The first dilution for which the percentage of positive cells is <20% is the most accurate limiting dilution for the vector. We suggest using the calculated titer from this dilution as the working titer.

3. 48 h later, examine the flask to determine cell confluence.
  a. Ideally, this should be ∼70%–80% at this time point.
4. Split HEK-293T cells.
  a. Aspirate all culture media.
  b. Add 10 mL cold DPBS to the flask and gently rinse the culture-side of the flask.
    **CRITICAL:** Take care not to dislodge adherent cells from the culture plate.
  c. Aspirate DPBS.
  d. Add 3 mL 0.05% Trypsin-EDTA into the flask and coat the culture-side of the flask.
  e. Wait 2–3 min, then wash the flask with 10 mL D10. Collect cells into a conical tube.
    **CRITICAL:** Thorough washing of the culture-side of the flask will ensure high-yield collection of all viable HEK-293T cells.

5. Centrifuge the cell suspension at 350 × *g* for 5 min.
6. Aspirate supernatant and resuspend the cell pellet in an appropriate volume of D10 for cell counting.
7. Perform a cell count and determine cell concentration.
  a. We anticipate that once recovered from thaw, 3 × 10^6^ HEK293T cells will yield ∼18–22 × 10^6^ cells after 48 h of growth in a T175 culture flask.
8. Add 3 × 10^6^ cells to each T175 flask and add D10 media to a final volume of 25 mL.
9. Place flask horizontally and rock the flask to ensure even cell distribution on the culture-side.
10. Incubate for 48 h.
  a. Repeat this process for at least 2 passages after thawing prior to initiating lentivirual production.
11. On the day before lentiviral production is to begin, plate 8 × 10^6^ HEK-293T cells in each T175 flask.
  a. This should result in ∼80% confluence 24 h later.
12. The following day, transfect HEK-293T cells with lentiviral packaging and CAR constructs:
  a. Thaw RSV.Rev, Gag/Pol, VSV-G, and target (i.e., containing the CAR transgene) plasmids.
  b. Combine 18 μg RSV.Rev, 18 μg Gag/Pol, 7 μg VSV-G and 15 μg target plasmid, per flask you intend to transfect, to a tube.
  c. Bring OPTI-MEM I media and Lipofectamine 3000 solution to room temperature (RT).
  d. For each transfection reaction, add 1.75 mL OPTI-MEM I each to two 5 mL round-bottom flow cytometry tubes.
  e. Add 87 μL Lipofectamine 3000 to one of two OPTI-MEM I-containing tubes.
  f. Add plasmid mix to the other OPTI-MEM I-containing tube.
  g. Add 116 μL of P3000 Enhancer Reagent to the tube containing the plasmid dilution and incubate for 5 min at RT.
    **CRITICAL:** Adding the P3000 Enhancer Reagent after the addition of plasmids is essential. Addition of plasmids after the P3000 Enhancer Reagent will result in immediate precipitation of DNA, thus reducing the transfection efficiency dramatically.
  h. Gently combine the contents of both flow cytometry tubes to produce the final transfection mixture and incubate for 15 min at RT.
  i. Position the flask of HEK-293T cells upright, then add the mixture to the bottom of the flask. Gently rock the mixture over the cells to ensure even distribution of this transfection mixture.
    ***Note:*** Adding the transfection mixture directly onto the cells can result in dislodging. This can reduce the number of vector-producing cells.
  j. Incubate for 24 h.
13. After 24 h, harvest and concentrate lentivirus from flasks:
  a. Pre-cool the tabletop centrifuge to 4°C and pre-warm D10 media.
  b. Collect media from each flask into a 50 mL conical tube.
  c. Add 25 mL fresh D10 media back to flasks for collection at the 72-h time point.
  d. Centrifuge the tube of collected media containing unconcentrated lentivirus at 350 × *g* for 5 min.
    ***Note:*** This step removes larger cell debris.
  e. Remove tubes from the centrifuge.
  f. Transfer the supernatant into 150 mL vacuum filter units with a 0.45 μm pore size.
  g. Attach to a vacuum.
    ***Note:*** This step removes smaller cell debris.
    ***Note:*** This filtration step can also be performed using 60 mL syringes with 45 μm syringe filters attached. This may be preferred if preparing several different constructs, where the volume of each individual preparation is low. If using this method, remove the syringe plunger, attach the filter, add supernatant into the syringe barrel, place syringe and filter on top of a 50 mL conical tube and carefully replace the plunger. Using gentle pressure, filter the supernatant into the conical tube.
  h. Distribute the filtered supernatant into new 50 mL conical tubes.
  i. Use a marker to mark the side of the tube and tube cap facing outward.
    ***Note:*** this indicates where the pellet should be:
  j. Centrifuge overnight at 8,000 × *g* at 4°C with acceleration on maximum and deceleration reduced to a low level (i.e., brake set at 3 out of 9).
    ***Optional:*** Overnight centrifugation can be replaced by ultracentrifugation for 2 h at 25,000 × *g*, under vacuum, at 4°C with maximum acceleration and reduced deceleration. This option requires distinct tubes for collection, depending on the specific ultracentrifuge.
  k. Towards the end of centrifugation, tightly pack ice into a bucket and create “slots” to hold 50 mL conical tubes, by quickly and forcefully plunging a 50 mL conical into the ice.
    ***Note:*** This creates a stable position for the tubes, minimizing disruption of the viral pellet.
  l. When the centrifuge stops, place the 50 mL conical tubes into the slots created on ice.
    **CRITICAL:** Be very careful not to disturb or dislodge the pellet.
  m. Aspirate supernatant carefully until there is approximately 0.5–1 mL of supernatant left in the tube.
  n. Resuspend the viral pellet in the remaining media.
    ***Note:*** Sometimes, a faint pellet can be seen on the marked side, but not always. We have not found this to correlate with lentiviral quality or quantity. Regardless, make sure to thoroughly wash the side of the tube where the pellet should be (identified by the mark made in step 12f) to ensure that the maximal quantity of lentivirus is collected. We recommend washing 120° (∼1/3) of the tube circumference and ∼2 cm above the expected pellet site.
  o. Set aside 70 μL of the concentrated virus in a cryotube.
    i. This will be used for viral titration.
  p. Distribute the remainder into the desired number of aliquots.
  q. Flash-freeze on dry ice.
  r. Store the virus aliquots at −80°C.
  s. Repeat steps 13a–r at the 72-h collection time point.
    **Pause point:** Virus can be stored at −80°C.

14. Thaw or purify the appropriate number of T cells required for lentiviral titration.
  a. Each viral collection being titered requires 0.7 × 10^6^ T cells.
15. Resuspend T cells at a concentration of 1.25 × 10^6^ cells/mL in R10.
16. Add to an appropriate culture vessel.
17. Wash the required number of anti-CD3/CD28 DynaBeads three times with R10:
  a. Calculate the number of beads required. Bead stimulation is optimal at a ratio of 3 beads per cell (3:1).
    **CRITICAL:** DynaBeads often come at a concentration of 400 × 10^6^ beads/mL. These beads are also heavy. As such, it is essential to ensure thorough resuspension before removing beads for use.
  b. Transfer the total amount of beads required into a 1.5 or 2 mL microcentrifuge tube containing 1 mL of R10.
  c. Place the microcentrifuge tube onto the DynaMag-2 magnet, or equivalent.
  d. Incubate for approximately 30 s until the beads have collected on the side of the tube closest to the magnet.
  e. Keep the tube on the magnet and aspirate the media.
    **CRITICAL:** being carefully not to dislodge the beads.
  f. Remove the microcentrifuge tube from the magnet.
  g. Resuspend the beads in fresh R10 media.
  h. Repeat 17b-g twice more to achieve a total of 3 washes.
    **CRITICAL:** Proper washing of the beads is important to remove the sodium azide present in the bead solution. Insufficient washing may result in poor cell viability.
  i. After third wash, resuspend the beads in an appropriate volume of R10 such that resuspension of the T cells in the bead suspension results in a final concentration of the 1 × 10^6^ T cells/mL.
  j. Mix beads with T cells and culture at 37°C, overnight.
    ***Note:*** Allow T cells to be stimulated for at least 18 h before proceeding. Bead–cell clumps can be observed under a light microscope.
  k. Thaw the 70 μL lentiviral titration aliquot.
  l. Set up a dilution plate by serially diluting the virus at a 1:3 dilution, 6 times resulting in a dilutions ranging from 1:3 to 1:729:
    i. In a U-bottom 96-well plate, add 100 μL R10 into wells A to F (6 wells) of a column, for each vector being titrated.
    ii. Add 50 μL of vector to well A and mix well by pipetting.
    iii. Transfer 50 μL from well A to well B in the same column.
    iv. Continue this serial dilution all the way down to the well F, which should result in a final volume of 150 μL in well F.
      ***Note:*** We use T cells (1:1 ratio CD4:CD8) for lentiviral titration. Although more labor intensive, titering virus on the same cell type to be used in experimental studies is ideal. We have compared titrations performed on primary human T cells and the T cell acute lymphoblastic leukemia (T-ALL) Jurkat cell line and found that titers are ∼10–20× higher when performed on Jurkat cells. It is feasible to determine titers on Jurkat cells with the knowledge of this discrepancy, but ultimately, the transduction efficacy in primary T cells is more unpredictable if using this method.
  m. Set up a titer plate by adding 100 μL of stimulated T cells (approximately 0.1 × 10^6^ cells) to each well of a U-bottom 96-well plate, mirroring the dilution plate.
  n. Add 100 μL of T cells to three additional wells (Figure 1, step 4).
    i. These will serve as non-transduced controls.

### Part II: CAR T cell production

**Timing: 16–18 days**

In this step, human T cells will be engineered to express CAR.

18. Purify or thaw previously purified T cells (we start with CD4 and CD8 cells at 1:1 ratio).

***Note:*** If using fresh T cells, proceed directly to stimulation (step 19). If using frozen T cells, we recommend resting thawed cells overnight in R10.

19. When ready to stimulate, wash the number of beads required to obtain a 3:1 ratio of beads:cells as previously described in steps 17a–h.
20. Mix T cells with beads to a final concentration of 1 × 10^6^ T cells/mL of R10.
21. Culture overnight at 37°C.
22. The next day, thaw sufficient virus to ensure a multiplicity of infection (MOI) of ∼3.
23. Add lentivirus to bead-stimulated T cells and mix gently.
24. Incubate for a further 5 days (for a total of six days of stimulation).
  a. Count cells on alternate days to trend growth.
25. Remove beads by magnetic separation as described in step 17s.

***Note:*** We also collect measurements of cell size, quantified as cell volume using a Coulter Principle counter. This additional parameter facilitates estimation of when T cells have plateaued from stimulation and have begun to rest. Not all cell counters allow this measurement, in which case, relying on the rate of T cell expansion (based on cell counts) should be sufficient.

26. Evaluate cells by flow cytometry-based detection of the CAR transgene to determine the transduction efficiency. Refer to troubleshooting, problem 1 if facing difficulties with transduction efficiency.
27. Freeze cells when expansion plateaus (i.e., where the rate of division is < 1.2× over two days) and the cell volume has decreased to <300 femtoliters.

***Note:*** If comparing multiple CAR or cell products to each other, cryopreservation will ensure a complete return to resting phase prior to tumor challenge. This serves to set an equal “starting point” for the cell products being compared in down-stream functional evaluations.

### Part III: Chronic stimulation of CAR T cells to induce dysfunction

**Timing: 13–17 days**

In this step, CAR+ T cells will be persistently exposed to antigen-expressing tumor cells to induce chronic receptor stimulation that drives the development of T cell dysfunction.

***Note:*** We routinely use Nalm6 cells that have been edited to disrupt the BH3 Interacting Domain Death Agonist (*BID*). These cells are partially-resistant to CAR T cell killing,^1^ facilitating chronic antigen–receptor engagement.

28. Thaw tumor cells in R10.
  a. Culture for at least four days to ensure recovery.
29. Thaw CAR T cells in R10 media and rest overnight at 37°C.
30. The day after thawing CAR T cells, count both tumor and CAR T cells.
31. Adjust both to a concentration of 1 × 10^6^ cells/mL in R10.
32. Establish co-cultures of 1 CAR+ T cell per 8 Nalm6 cells (1:8 effector:target (E:T) ratio).

***Note:*** While the absolute cell numbers can vary depending on cell availability, we aim for 0.25–2 × 10^6^ T cells per culture. Each co-culture condition is set up in triplicate.

33. Two days after establishing co-cultures, collect 50–100 μL sample into a flow cytometry tube or a U-bottom 96-well plate.
34. Stain cells to determine the number of CAR T cells (using either an antibody directly targeting the CAR or a marker contained within the CAR vector) and the number of Nalm6 cells (our Nalm6 cells are engineered to express GFP, but alternatively you could use CD10 or CD20, which are robustly expressed by Nalm6). Refer to troubleshooting, problem 2 if facing difficulties with this step.

***Note:*** Throughout this protocol, check culture plates daily and replenish media when necessary, as determined by media color and approximate cell density. We aim to maintain cell cultures at 0.25–2 × 10^6^ cells/mL, as stated above.

***Note:*** Due to the targeting of CD19 by the CAR, quantifying CD19 surface expression can be difficult.

***Note:*** Antibodies targeting additional surface or intracellular proteins of interest can be included in this staining panel.

35. Analyze stained co-cultures by flow cytometry.

**CRITICAL:** Ensure that the stopping gate is set to a specific volume and not to a specific event count. This enables calculation of cell concentration, and thus total cell number, in the culture well.

***Note:*** Alternatively quantification beads (such as the CountBright Absolute Counting Beads from Thermo Fisher) can be used to determine cell counts during flow cytometry analysis.

36. Use the following formula to calculate the number of cells within each culture:
  a. (cell count by flow cytometry/volume of cells collected for analysis) × resuspension volume for analysis × correction factor.
  b. For example: Each co-culture contains 1 mL and 50 μL was removed for analysis. This was then resuspended in a final analysis volume of 200 μL, of which 50 μL was acquired on the cytometer. The total number of Nalm6 cells in the co-culture would be:

**Figure:**
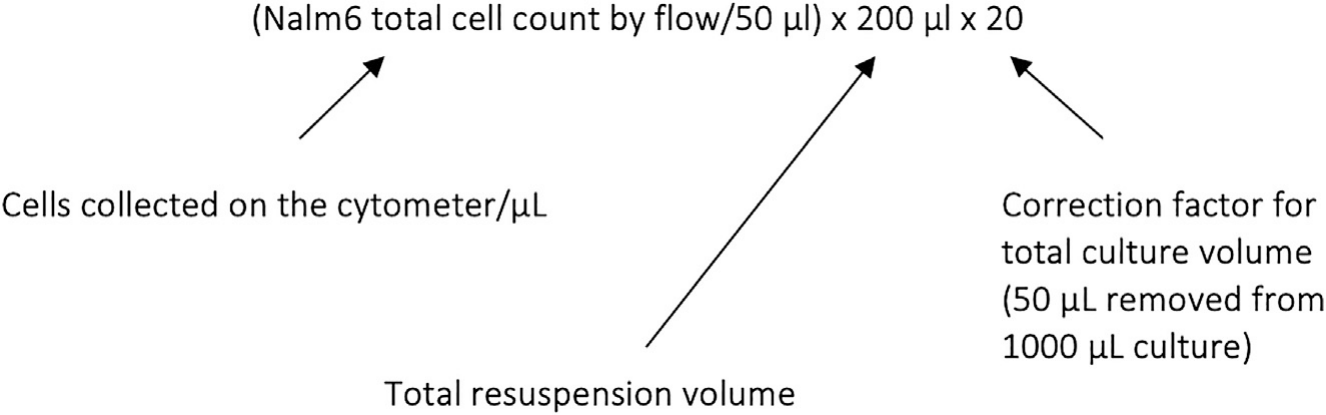

37. Calculate the total number of Nalm6 cells and CAR+ T cells in each co-culture and then determine the following three key measurements: Nalm6 fold expansion, CAR+ T cell fold expansion, E:T ratio.

**CRITICAL:** To perform these calculations effectively the culture volume of each well should be measured at each analysis time point.

38. Add an appropriate number of Nalm6 cells to each co-culture to re-establish an E:T ratio of 1:8,
  a. Adjust culture volumes to ensure equivalent cell concentrations in each well.

**CRITICAL:** It is important to consider the number of residual Nalm6 cells in each culture when adding fresh Nalm6 to re-establish the E:T ratio.

39. Repeat steps 33–38 every other day for the duration of the co-culture.

***Note:*** For standard CD28 and 4-1BB CAR T cells, we anticipate the development of dysfunction—as defined by a T cell fold-change of <1 and/or Nalm6 fold-change of >1—to occur after 13–17 days of co-culture.

***Note:*** As cultures expand, it may become infeasible to maintain the entire co-culture due to the quantity of Nalm6 cells required. T cells routinely expand >100× during these cultures, which would require >800 × 10^6^ Nalm6 cells per well for a co-culture that started with 1 × 10^6^ T cells. In these situations, we only maintain a fraction of the culture after each measurement. This mandates calculation of only fold-change in Nalm6 cells and T cells relative to the previous day. While non-traditional (compared to a calculation of fold-change over the duration of the co-culture), these measurement still provide the critical data required i.e., a fold-change in T cell counts of >1 demonstrating continued cell expansion, and similarly a fold-change in Nalm6 cells of <1 demonstrating continued killing.

### Part IV: Down-stream characterization of CAR T cells

To perform any directed analysis, we purify CAR T cells from co-cultures at various time points during chronic stimulation. This can be performed either by flow-based cell sorting using the same antibodies used to identify CAR+ T cells during co-culture monitoring, or by magnetic isolation based on a surface marker. Refer to troubleshooting, problem 3 for help if encountering difficulties.

***Note:*** At later time points, after the onset of T cell dysfunction and outgrowth of target cells, we recommend performing dead cell and target cell depletion prior to T cell purification regardless of purification method. This will significantly enhance the yield of CAR+ T cells. We routinely use the Dead Cell Removal Kit and CD10 Microbeads (both from Miltenyi Biotec) for these two steps.

## Expected outcomes

**Figure 2:**
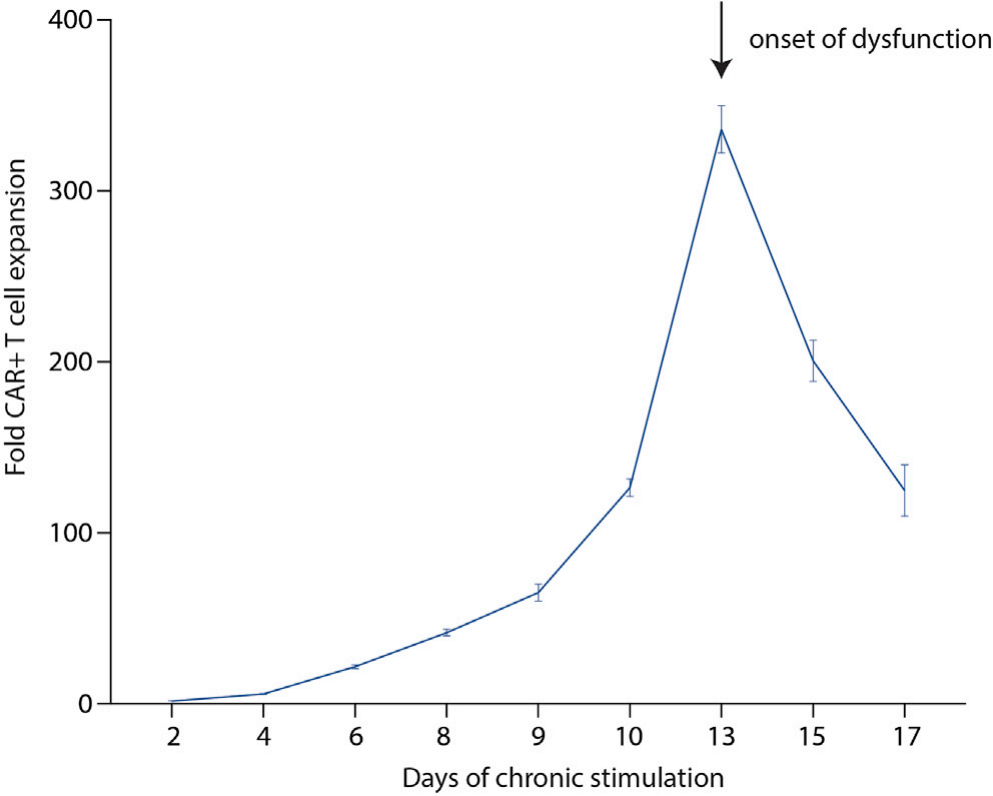
CAR T cell growth kinetics over the course of chronic stimulation

In the initial phase of chronic stimulation, CAR T cells will initially enact potent effector functions as observed in acute stimulation assays. This includes control of tumor cell growth despite recurrent re-addition of tumor cells and robust T cell expansion (Figure 2). However, after 10–13 days of chronic stimulation, T cells will lose their effector functions, resulting in an inability to control tumor cell growth and in T cell contraction.

## Limitations

This protocol has been optimized for assessment of human T cells engineered to express CD19-targeted CARs in co-cultures with CD19+ Nalm6 acute lymphocytic leukemia (ALL) cells. Other cell lines and/or T cells expressing other CARs will require titration and optimization of E:T ratios, frequency of re-establishing E:T ratios, and optimal T cell isolation times to enable consistent and comparable studies. In most studies, investigators will be interested in studying T cells after the onset of dysfunction. As shown in Figure 2, T cells undergo abrupt apoptosis after becoming dysfunctional. This limits both the number of cells available for analysis, but also the quality of these cells. It is vital to remove dead cells prior to any genomic, transcriptomic or bulk analyses. An efficient method of cell purification is essential for the success of down-stream T cell evaluations, as is a reliable flow cytometer. When comparing T cells with distinct metabolic behaviors it may be difficult to ensure similar composition of culture media as these cells undergo chronic stimulation. While we do our best to ensure equivalent culture concentrations throughout the protocol, differences in biochemistry may impact T cell biology that are independent of the primary hypotheses. Finally, as with all studies of primary human T cells, donor variability can result in significant difficulty comparing data across distinct experiments. For example, one donor’s T cells may expand 200-fold during chronic stimulation while another’s may expand 50-fold. We encourage comparison of trends as opposed to absolute quantitative results.

## Troubleshooting

### Problem 1

Low transduction efficiency (steps 18–26).

### Potential solution

Several factors can influence T cell transduction efficiency. Inter-donor variability can result in varied efficiency. The quality of antibody used for the detection of surface CAR expression (or marker expression) is also very important. Large volumes of lentiviral vector used for transduction can also impact efficiency of transduction, even with correction for target MOI. If recurrently encountering difficulty with transductions (across multiple donors with a validated antibody for detection), we would advise increasing vector concentrations after centrifugation. In addition, resequencing the target plasmid can aid in identifying potential mutations or disruptions in key sequences.

### Problem 2

Poor T cell effector function in co-culture assays (steps 28–34).

### Potential solution

As mentioned above, inter-donor variability can result in varied effector function *in vitro*. While we aim to establish cultures at a high E:T ratio of 1:8, some CAR T cell products are overwhelmed by this. This is particularly a problem at first encounter when T cells start at rest. We recommend dropping the E:T ratio for the first stimulation (down to 1:4 or 1:2) and monitoring cultures more closely (daily) to determine the right time to re-establish E:T ratios. In our experience, a few days at a lower E:T ratio allows T cells to activate effector functions and these cultures can be subsequently maintained at an E:T of 1:8 thereafter.

### Problem 3

Difficulty collecting sufficient T cells for down-stream analysis (Part IV).

### Potential solution

After the onset of dysfunction, CAR T cells can undergo apoptosis rather quickly. Further, these cultures become overwhelmed with rapidly-dividing cancer cell lines. Finding the right time to isolate T cells for down-stream studies can be difficult, and again, donor-dependent. Close monitoring of these cultures will allow investigators to identify a 1–2 day range when T cells begin to lose function. We recommend isolating T cells either on this day or within 24–48 h of this time point to maximize yield. As mentioned in the protocol, removal of dead cells and depletion of target cells will significantly enhance T cell yield. A highly-capable cell sorting facility or cell sorter is necessary if using a flow-based method of isolation. In our experience, magnetic sorting can be both more time-efficient and less harsh on cells, resulting in better cell viability following isolation. Lastly, establishing larger cultures to increase T cell yields at analyses time points is also an option.

## Resource availability

### Lead contact

Further information and requests for resources and reagents should be directed to and will be fulfilled by the lead contact, Nathan Singh (nathan.singh@wustl.edu).

### Materials availability

We have not generated any new reagents or materials as part of this protocol. We have several template Excel sheets to assist with co-culture calculations and are happy to share these, or any other materials, with interested investigators.

## Data Availability

We have not generated any new code in the development of this protocol.

## References

[1] Singh N., Lee Y.G., Shestova O., Ravikumar P., Hayer K.E., Hong S.J., Lu X.M., Pajarillo R., Agarwal S., Kuramitsu S. (2020). Impaired death receptor signaling in leukemia causes antigen-independent resistance by inducing CAR T-cell dysfunction. Cancer Discov..

[2] Singh N., Perazzelli J., Grupp S.A., Barrett D.M. (2016). Early memory phenotypes drive T cell proliferation in patients with pediatric malignancies. Sci. Transl. Med..

[3] Das R.K., Vernau L., Grupp S.A., Barrett D.M. (2019). Naive T-cell deficits at diagnosis and after chemotherapy impair cell therapy potential in pediatric cancers. Cancer Discov..

[4] Schultz L.M., Baggott C., Prabhu S., Pacenta H.L., Phillips C.L., Rossoff J., Stefanski H.E., Talano J.A., Moskop A., Margossian S.P. (2022). Disease burden affects outcomes in pediatric and young adult B-cell lymphoblastic leukemia after commercial tisagenlecleucel: a pediatric real-world chimeric antigen receptor consortium report. J. Clin. Oncol..

[5] Lynn R.C., Weber E.W., Sotillo E., Gennert D., Xu P., Good Z., Anbunathan H., Lattin J., Jones R., Tieu V. (2019). c-Jun overexpression in CAR T cells induces exhaustion resistance. Nature.

[6] Long A.H., Haso W.M., Shern J.F., Wanhainen K.M., Murgai M., Ingaramo M., Smith J.P., Walker A.J., Kohler M.E., Venkateshwara V.R. (2015). 4-1BB costimulation ameliorates T cell exhaustion induced by tonic signaling of chimeric antigen receptors. Nat. Med..

[7] Good C.R., Aznar M.A., Kuramitsu S., Samareh P., Agarwal S., Donahue G., Ishiyama K., Wellhausen N., Rennels A.K., Ma Y. (2021). An NK-like CAR T cell transition in CAR T cell dysfunction. Cell.

[8] Kutner R.H., Zhang X.Y., Reiser J. (2009). Production, concentration and titration of pseudotyped HIV-1-based lentiviral vectors. Nat. Protoc..

